# Recent Advancements of Bio-Derived Flame Retardants for Polymeric Materials

**DOI:** 10.3390/polym17020249

**Published:** 2025-01-20

**Authors:** Min Chen, Qinhe Guo, Yao Yuan, Ao Li, Bo Lin, Yi Xiao, Lulu Xu, Wei Wang

**Affiliations:** 1 Fujian Provincial Key Laboratory of Functional Materials and Applications, School of Materials Science and Engineering, Xiamen University of Technology, Xiamen 361024, China; chanamanam@163.com (M.C.); blwdlx@163.com (Q.G.); 2020000004@xmut.edu.cn (Y.X.); 2 School of Mechanical and Manufacturing Engineering, University of New South Wales, Sydney, NSW 2052, Australia; ao.li@unsw.edu.au (A.L.); bo.lin@unsw.edu.au (B.L.); 3 School of Chemical Engineering, University of New South Wales, Sydney, NSW 2052, Australia; lulu.xu1@unsw.edu.au

**Keywords:** bio-derived flame retardant, polymer, sustainable, renewable, flame retardancy

## Abstract

The sustainable flame retardancy of polymeric materials is a key focus for the direction of the next generation in the field of fire safety. Bio-derived flame retardants are gaining attention as environmentally friendly additives due to their low ecological impact and decreasing costs. These compounds can enhance char formation in polymeric materials by swelling upon heating, attributed to their functional groups. This review explores various biomolecules used as flame retardants, including phytic acid, chitosan, lignin, tannic acid, and bio-derived phosphorus and nitrogen compounds, emphasizing their flame-retardant properties and compatibility with different polymer matrices. The primary focus is on the structural characteristics, modifications, and flame-retardant behaviors of these bio-derived additives, particularly regarding their mechanisms of action within polymeric materials. Finally, the review explores the opportunities, current challenges, and future directions for the practical application of bio-derived flame retardants in polymer materials.

## 1. Introduction

Polymeric materials are widely utilized in everyday life because of their versatility, lightweight nature, and simple processing [1,2]. They are essential in applications across construction, transportation, textiles, electronics, and consumer goods, contributing significantly to modern technological advancements and convenience. Despite their numerous advantages, the inherent structural characteristics of polymers present notable fire hazards, primarily due to their organic composition [3,4,5,6,7,8]. The molecular structure of most polymers, rich in carbon and hydrogen atoms, acts as a potent fuel source, facilitating rapid combustion and significant heat release [9,10]. Additionally, the low thermal conductivity of these materials leads to localized heat accumulation, further accelerating the spread of flames. Such properties not only pose safety risks but also contribute to severe environmental and economic consequences during a fire [11,12,13]. As the use of polymers continues to expand, developing innovative approaches to mitigate these fire hazards has become a critical focus in material science and engineering, particularly through the incorporation of flame-retardant technologies and modifications.

Preventing fire hazards involves a comprehensive approach that incorporates fire protection measures, such as smoke detectors and sprinklers, alongside the development and application of materials designed to minimize ignition and suppress the spread of fire [14,15]. Among these strategies, the application of flame retardants (FRs) plays a vital role in enhancing the fire safety of materials, particularly polymeric materials that are prone to combustion due to their organic nature. FRs are categorized into several categories according to their chemical composition, including halogenated, phosphorus-based, nitrogen-based, and inorganic types [16,17,18,19,20]. Each category is characterized by the specific chemical elements or compounds that form the core structure of the flame retardant, which influences its effectiveness and mechanism of action in suppressing combustion. Phosphorus-based FRs function primarily by promoting the formation of protective chars during combustion, which helps to insulate the material and inhibit the progress of the fire. The efficiency with which phosphorus-based FRs exert their flame-retardant effects is strongly influenced by the level of oxygenation at the phosphorus atom [21,22,23,24,25]. Nitrogen-based FRs, such as melamine, triazine and its derivatives, are particularly effective in combination with phosphorus compounds, creating cooperative effects that improve fire resistance [26,27,28]. Halogenated FRs, which contain bromine or chlorine, are highly effective in the gas phase but have raised environmental and health concerns because of the harmful emissions produced during combustion [29,30]. Flame-retardant agents can be added to polymer materials in two main ways: physical blending or chemical modification. In physical blending, flame retardants, such as powders, granules, or fibers, are mixed into the polymer without changing its chemical structure. These agents are dispersed in the polymer during processing methods like melt processing or solution blending to improve flame resistance. However, ensuring uniform dispersion and preventing the migration or leaching of the FRs in the polymers remains a challenge. In chemical modification, the FR molecules are chemically attached to the polymer chain through processes like grafting or copolymerization. This method integrates the flame retardant directly into the polymer structure, providing stronger and longer-lasting flame resistance, improved thermal stability, and preventing the agents from migrating or leaching out. However, the process is often intricate and involves specialized chemical reactions, such as grafting or copolymerization, which can lead to increased production time and costs.

Given the toxicity and environmental hazards posed by conventional flame-retardant materials, there is an increasing need to minimize their widespread use [31,32]. This has driven significant interest in the development of driven, non-toxic flame-retardant materials that can provide effective fire protection while ensuring safety for both humans and ecosystems. From an environmental perspective, the focus has shifted toward utilizing renewable and sustainable resources as alternatives [33,34,35,36,37]. Renewable materials, sourced from natural origins like bio-based polymers, lignin, cellulose, and other organic compounds, offer the dual advantage of reducing reliance on petrochemical-based products and mitigating environmental pollution [38,39,40]. These materials not only conform to the principles of green chemistry but also hold promise for achieving effective flame retardancy through innovative design and functional modifications. The development of such sustainable FRs addresses the urgent demand for fire protection solutions that are both safer and more environmentally friendly.

Bio-derived FRs are made from biomolecules sourced from natural materials such as cellulose, chitosan, lignin, phytic acid, and others. These biomolecules have garnered considerable attention as environmentally friendly FRs because of their inherent non-toxicity, high biocompatibility and renewability [41,42,43]. Unlike traditional FRs, which often involve halogenated compounds and pose risks to human health and environments, bio-derived FRs align with the principles of sustainability and green chemistry. Their capacity to improve flame retardancy stems from functional groups in their molecular structures, such as hydroxyl, carboxyl, and phosphate groups, which contribute to char formation and inhibit combustion processes [44]. Additionally, the utilization of biomolecules can reduce the environmental footprint of flame retardants, offering a safer and more sustainable alternative for fire protection in various applications. With the increasing demand for eco-friendly materials, bioderived flame retardants offer a promising solution for achieving both effective and sustainable fire protection [45].

Bio-derived FRs contain crucial components that are conducive to char formation, which plays a key role in inhibiting combustion. However, despite these beneficial characteristics, the flame retardancy of bio-derived FRs is often insufficient when used alone, limiting their effectiveness in certain applications [46,47]. As a result, a range of modification strategies is needed to enhance the flame resistance of these materials. This paper offers a thorough review of recent research advancements in biomass-based FR additives, which are gaining prominence and show significant potential for broad applications. Specifically, the review will focus on research progress in areas involving cellulose, chitosan, lignin, starch, phytic acid (PA), and tannic acid (TA) as key materials. The discussion centers on bio-derived functionalized products as effective FRs, elaborating on modification strategies, fire safety performance, optimization techniques and life cycle assessment (LSA) analysis. Finally, the paper will also explore the opportunities and challenges associated with the application and advancement of bio-derived FRs.

## 2. Flame-Retardant Mode of Action

Flame retardants play a crucial role in decreasing the flammability of polymers by delaying combustion or, in some instances, preventing ignition altogether. These mechanisms are generally divided into two main types: the condensed-phase mechanism and the gas-phase mechanism [48,49,50]. Each of these mechanisms plays a critical role in inhibiting combustion, but they operate in fundamentally different ways, affecting different stages of the fire process.

Flame retardancy of materials is typically measured using tests like the cone calorimeter, limiting oxygen index (LOI) and UL-94 rating. The cone calorimeter is a widely used experimental method for evaluating the fire behavior and flame-retardant properties of materials. It provides detailed information on how a material reacts to heat and fire by measuring key parameters such as peak heat release rate (pHRR), time to ignition (TTI), total heat release (THR), and smoke production. The LOI test determines the lowest oxygen concentration required to sustain the combustion of a material. A higher LOI means better flame resistance. The UL-94 rating evaluates how a material reacts to an open flame, checking how quickly it extinguishes and whether it drips flaming material. These tests are essential for assessing fire safety in materials, especially in industries where flame resistance is important. In the UL94 standard, V0 and V1 are classifications that evaluate the flammability of plastic materials by evaluating their tendency to extinguish after being exposed to a flame. A V0 rating represents the highest level of flame resistance, indicating that the material extinguishes the flame within 10 s after the flame is removed, without dripping molten material that could ignite a cotton swab placed below the specimen.

**Figure 1:**
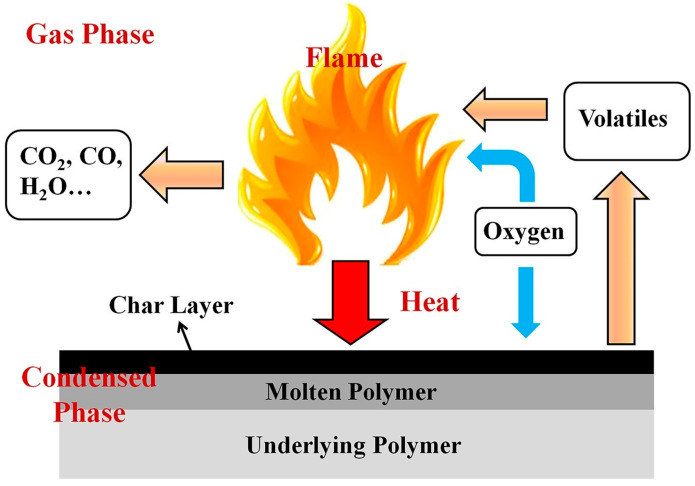
Illustration of model for flame-retardant actions [51]. Copyright 2017. Reproduced with permission from Elsevier Science Ltd.

A model of flame retardancy is presented in Figure 1, which provides a detailed representation of the different zones involved in the combustion process of a polymer material [51]. The model includes several key zones: the flame zone, the char layer, the molten polymer, and the underlying polymer. Each of these zones plays a distinct role in the material’s response to fire and the effectiveness of FR mechanisms. The condensed-phase mechanism works at the material’s surface. When subjected to heat, FRs in this phase facilitate the development of a stable, carbon-enriched char layer. These chars function as protective layers, shielding the underlying material from further heat and oxygen, thus preventing it from reaching the high temperatures required for combustion. Furthermore, the char layers can reduce the emission of combustible gases and restrict overall heat transfer.

The gas-phase mechanism generally works by disrupting the combustion process, interfering with the reaction of volatile pyrolysis products emitted from the polymer. During combustion, polymers degrade into various volatile compounds, such as hydrocarbons and carbon monoxide. These volatile gases can react with oxygen, sustaining the flame. Flame retardants that operate through the gas-phase mechanism act by forming radicals or other reactive species that interact with these volatile compounds, preventing the formation of highly flammable gases or inhibiting their reaction with oxygen. This interference reduces the overall heat release rate, thereby aiding in the control or suppression of fire spread.

**Figure 2:**
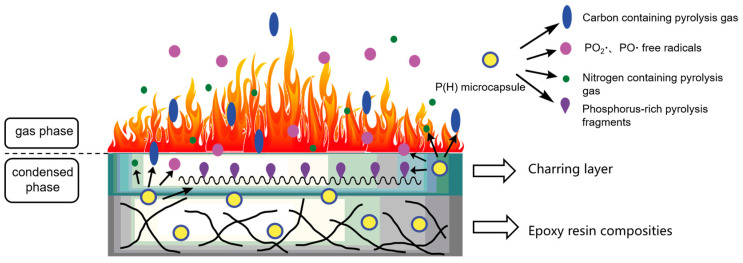
Flame retardancy mechanism of phosphorus FR in the composite [52]. Copyright 2020. Reproduced with permission from Elsevier Science Ltd.

A common strategy for designing flame-retardant materials is the hybrid mechanism, where two or more flame-retardant agents with different mechanisms are combined. For instance, phosphorus-based FRs are effective in both condensed-phase mechanism and the gas-phase mechanism. Figure 2 illustrates the mechanism by which phosphorus-based FRs function in the composite [52]. In the gas-phase mechanism, phosphorus compounds interfere with the chemical processes occurring in the flame, slowing combustion by inhibiting the formation of flammable gases and reducing heat release. Meanwhile, in the condensed-phase mechanism, phosphorus-based FRs promote the formation of a char layer on the polymer surface, forming physical barriers that shield the underlying material from heat and oxygen. This combined action greatly enhances fire resistance. Additionally, boron derivatives improve the cohesion and durability of the char, suppressing the release of flammable volatiles and limiting fire propagation, with these effects varying based on the specific type of boron compound used [53]. In contrast, metal oxides exhibit flame resistance through a variety of mechanisms, including catalytic action in the decomposition of polymer matrices and the promotion of crosslinked structures at elevated temperatures. Specific metal oxides, such as cuprous oxide [54] and manganese dioxide [55], also facilitate smoke suppression by reducing the generation of toxic combustion byproducts. These mechanisms, often synergistic with other flame-retardant additives, contribute to their widespread application in polymeric materials.

## 3. Progress in the Typical Bio-Derived Flame Retardants

### 3.1. Cellulose-Derived Flame Retardants

Cellulose is a promising bio-derived source for FRs due to its abundance and renewability [43,56], which is primarily derived from plants and is produced on a massive scale, with an estimated annual global production ranging from 10^9^ to 1.5 × 10^12^ tonnes [57]. Cellulose consists of two distinct structural domains: an amorphous region and a crystalline region. Crystalline cellulose features a highly ordered structure where the individual cellulose chains are aligned in a parallel arrangement and tightly packed through hydrogen bonds. On the other hand, amorphous cellulose has a more disordered arrangement of chains, resulting in a structure that is more flexible and exhibits important viscoelastic properties [58]. Its widespread availability, biodegradability, and structural versatility make cellulose an attractive candidate for sustainable flame-retardant applications.

Cellulose, as a typical natural polymer, is widely utilized as a filler in polymeric materials. Its inherent hydrophilicity promotes interactions with other components in composite materials. Additionally, cellulose exhibits higher heat resistance compared to many other natural polymers, enhancing the thermal stability of composites. Its non-toxic nature ensures safety for human health and the environment, while its hygroscopic property allows it to absorb moisture, which can influence the performance of material in various conditions. Meanwhile, nanocellulose has become recognized as an effective and sustainable material in flame-retardant applications. Nanocellulose exists in several forms, including cellulose nanocrystals (CNC), cellulose nanofibers (CNF), and bacterial cellulose (BC), each with distinct characteristics that enhance their flame resistance potential [59,60]. Despite being combustible, cellulose can be treated to enhance its resistance to fire, making it a versatile and sustainable choice for enhancing the functionality of polymer composites.

Grafting cellulose with flame-retardant agents, such as phosphorus, boron, silicon, or nitrogen, significantly enhances its flame resistance by improving the formation of a protective char layer during combustion, which is critical in slowing the spread of flames and limiting the intensity of the fire. Phosphorus compounds can be chemical grafted onto the cellulose backbone and the incorporation of phosphorus introduces char-forming and flame-retardant capabilities [61,62]. Boron-based flame retardants help create stable, cohesive chars that insulate the material from high temperatures, while silicon compounds enhance thermal stability and resistance to decomposition. Nitrogen-containing flame retardants can further contribute to flame inhibition by releasing non-flammable gases during combustion, which helps to dilute the combustion atmosphere and suppress the fire. The combination of these elements not only enhances the structural integrity of cellulose during fire exposure but also leads to cooperative effects that significantly improve its flame-retardant performance. Guo et al. developed polylactic acid (PLA) composites incorporating resorcinol bis (diphenyl phosphate) (RDP) and cellulose [63]. While the inclusion of RDP typically led to the degradation of PLA and compromised its mechanical properties, the study revealed that adding cellulose counteracted this effect, enhancing the mechanical strength of PLA in the presence of RDP. Additionally, the researchers demonstrated that the PLA composite containing both RDP and cellulose successfully achieved the UL-94 V-0 rating, indicating excellent flame-retardant performance.

### 3.2. Chitin/Chitosan-Derived Flame Retardants

Chitosan, a water-soluble, semi-crystalline polymer that is water-soluble in acidic conditions, is derived from the deacetylation of chitin, which is present in the exoskeletons of crustaceans and the cell walls of fungi [64,65]. Chitin, a natural biopolymer found in the exoskeletons of arthropods and insects, has gained attention as a potential flame-retardant material due to its unique structure and chemical properties. Zhang et al. [66] prepared bio-based nanopapers using deacetylated chitin nanocrystals (ChNCs) and phosphorylated cellulose nanofibers (P-CNFs) through electrostatic complexation. The nanopapers demonstrated good thermal stability, with a 61% reduction in the pHRR upon incorporating 30 wt.% P-CNF, showing excellent self-extinguishing properties and enhanced fire resistance. Zhang et al. [67] used chitin nanocrystals and MXene to create a sustainable, flame-retardant biofilm through a simple evaporation-induced self-assembly process. The resulting biofilm showed a 90.1% reduction in pHRR. As the second most abundant natural polysaccharide after cellulose, chitosan has attracted significant attention for its renewable nature, biodegradability, and functional versatility. Its unique molecular structure, featuring amino groups, enables excellent reactivity and makes it highly versatile across various applications. Its natural origin, coupled with excellent film-forming and adhesive properties, makes it an ideal candidate for sustainable material development. Chitosan’s ability to form strong interactions components enhances the mechanical properties and structural integrity of composites, while its biodegradability ensures minimal environmental impact. Additionally, its inherent antimicrobial and flame-retardant characteristics further expand its applicability in advanced composite materials. These attributes have made chitosan an essential element in the pursuit of eco-friendly and high-performance bio-sourced materials.

Chitosan stands out for its ability to promote char formation during combustion, enhance thermal stability, and contribute to the release of non-flammable gases. These properties, combined with its compatibility with various polymer matrices, make chitosan a promising bio-based flame retardant for environmentally friendly fire protection strategies. Studies on flame retardants after grafting chitosan have demonstrated significant improvements in its fire resistance properties. Researchers have explored various grafting techniques, including the incorporation of phosphorus, boron, and nitrogen-based flame-retardant agents onto chitosan. Wang et al. [68] synthesized a boron-containing chitosan-based compound, HBS, and incorporated it into epoxy resin (EP) to enhance its flame retardancy. The cooperative effects between the boron-containing chitosan-based compound HBS and ammonium polyphosphate (APP) significantly enhanced the flame retardancy of EP composites. When combined in a 1:3 weight ratio at a total addition of 10%, the composite (labeled S3) achieved a UL-94 V-0 rating, indicating excellent flame resistance. The limited oxygen index (LOI) value of S3 (30.5%) was notably higher than that of unmodified EP (22.6%) and EP modified with 10% APP (26.1%). The modified EP composites exhibited a 51.5% reduction in pHRR and a 33.5% reduction in THR compared to the unmodified EP. This improvement is attributed to the combination of bio-based flame retardants (HBS) and traditional ones (APP), which form dense, durable char layers on the EP surface during combustion. The char layers effectively reduce heat transfer, limit combustible gas production, and suppress smoke emission, thereby enhancing fire resistance and minimizing heat and smoke release. The cooperative effects of HBS and APP significantly enhance fire resistance and safety.

**Figure 3:**
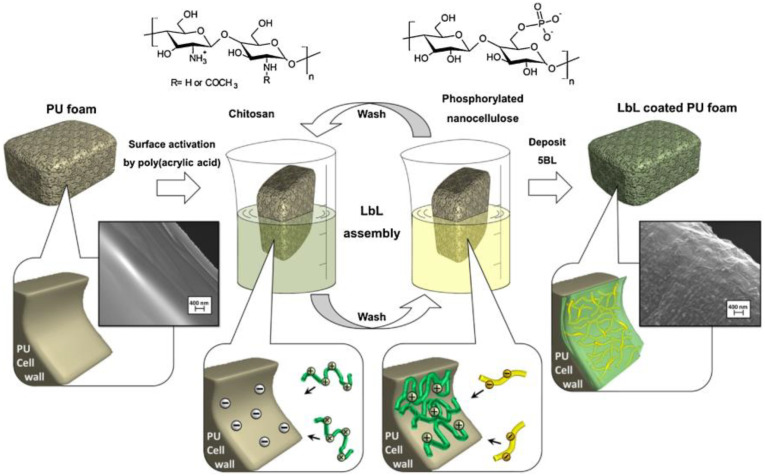
Schematic representation of the LbL process [70]. Copyright 2018. Reproduced with permission from Elsevier Science Ltd.

Zhang et al. [69] employed chitosan, Fe_3_O_4_, and halloysite nanotubes (HNT) to develop a three-dimensional network structure, effectively improving the flame resistance of EPs. In this system, chitosan serves as a carbon-forming agent, promoting the catalytic carbonization of the polymer, while Fe_3_O_4_ on the HNT surface further enhances the composite’s carbonization efficiency. Additionally, the incorporation of HNT@chitosan@Fe_3_O_4_ significantly decreases the release of toxic volatiles during the combustion of EPs. As shown in Figure 3, Carosioa et al. [70] employed a layer-by-layer assembly method to apply chitosan and phosphorylated cellulose nanofibers (P-CNF) onto polyurethane (PU) foam. Incorporating just 8 wt.% chitosan/P-CNF resulted in a 31% reduction in the composite’s pHRR and effectively suppressed droplet formation during combustion.

### 3.3. Lignin-Derived Flame Retardants

Lignin, a natural aromatic polymer, is one of the three primary components of wood, alongside cellulose and hemicellulose [38,39,71,72]. As a complex and highly crosslinked macromolecule, lignin serves a critical structural role in plants, providing rigidity, resistance to degradation, and acting as a natural barrier against pathogens. Its abundance, renewable nature, and aromatic structure make lignin an attractive candidate for various applications, including its potential use as a bio-based FR.

As demonstrated in Table 1, lignin and its derivatives are commonly utilized to enhance fire safety. The potential of lignin as a FR in non-charring polypropylene (PP) was investigated by De Chirico et al. [73]. Their study revealed that incorporating lignin with phosphate compounds and aluminum hydroxide enhanced the thermal degradation time, extended combustion duration, and improved the char yield of PP, achieving a 6 wt.% char yield with 15 wt.% lignin. Song et al. [74] studied the flame retardancy of acrylonitrile butadiene styrene (ABS) enhanced with kraft lignin. Incorporating lignin improved the flame-retardant properties of ABS by lowering the heat release rate (HRR) and shortening the ignition time. Xu et al. synthesized a lignin-based flame retardant through a co-precipitation process involving sodium lignosulfonate and layered double hydroxide (LDH), achieving improved dispersion within the polypropylene (PP) matrix. Consequently, the flame-retardant PP exhibited a 62.9% decrease in the pHRR compared to the unmodified PP, highlighting the superior flame retardance of the lignin-based material.

### 3.4. Phytic Acid-Derived Flame Retardants

Phytic acid (PA), also referred to as inositol hexakisphosphate, is a naturally occurring organic compound characterized by its highly phosphorylated structure [90,91,92]. It consists of an inositol ring with six phosphate groups and twelve hydroxyl groups, endowing it with strong chelating and reactive properties. Moreover, PA acts as an acid source in intumescent flame-retardant systems, and its high phosphorus content enhances char formation and helps stabilize the residual carbon. However, its direct application has been found to negatively impact the mechanical properties of cotton fabrics, primarily due to its acidic nature, which can degrade cellulosic fibers [93]. Table 2 summarizes recent studies on the use of PA as flame retardants.

A novel bio-based flame retardant, calcium magnesium phytate (CaMg-PA), along with acid-treated carbon nanotubes (CNT), was used to enhance the flame retardancy of PLA [94]. When 19 wt.% CaMg-PA and 1 wt.% CNT were incorporated into PLA, the pHRR of the composite reduced from 385 kW/m^2^ in the pure sample to 249 kW/m^2^. The addition of these flame retardants also reduced smoke emission and increased carbon residue during combustion. The combined use of both flame retardants resulted in superior flame retardancy compared to the individual use of either component.

**Figure 4:**
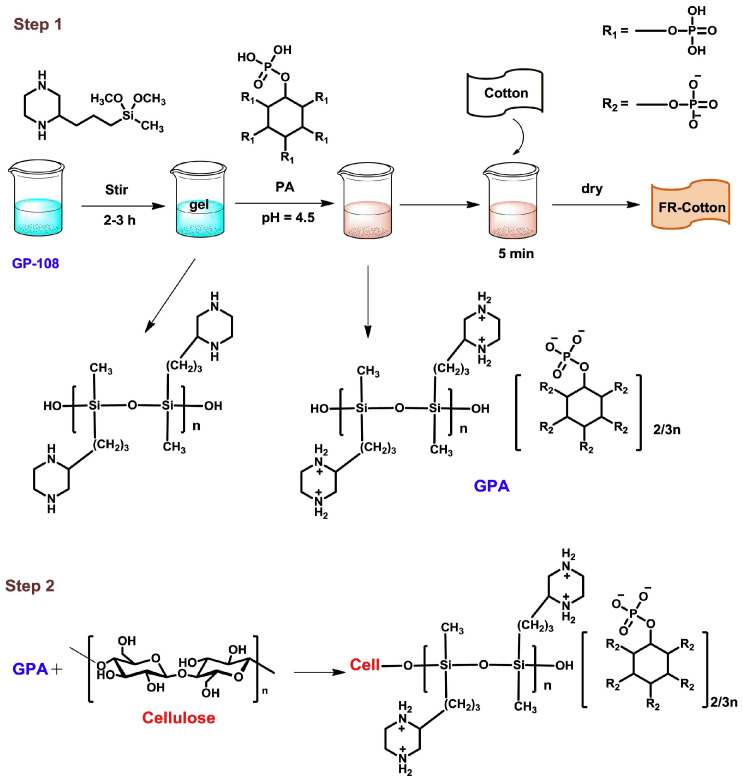
Flame retardancy mechanism of phosphorus FR in the composite [95]. Copyright 2019. Reproduced with permission from American Chemical Society.

As previously mentioned, the strong acidic nature of PA limits its direct use as an FR. To overcome this limitation, a gel of (3-Piperazinylpropyl)-methyldimethoxysilane (GP-108) was prepared using a sol-gel process and then reacted with PA to create an FR mixture with reduced acidity [95]. As illustrated in Figure 4, flame-retardant cotton fabric was developed using the GPA mixture through a dip-coating method. The resulting GPA-based FR was effectively grafted onto the fabric’s surface, and the cooperative effect was found to be positively correlated with the loading of GP-108, leading to increased char yields and an improved LOI. The flame retardancy mechanism involved the release of non-flammable NH_3_ gas from the -NH_2_^+^ group in GP-108 and PA, which catalyzed char formation during combustion.

**Table 2:** Comparison of LOI and UL-94 test results for flame-retardant composites with different phytic acid systems.

Polymer Matrix	FR	Fillers Content	pHRR Reduction (%)	LOI (%)	UL-94 Rating	Published Year	Ref.
PP	PA-Zn	2 wt.%	-	19.2	N.R.	2017	[96]
PP	IFR/PA-Zn	19 wt.%	64.0	29.2	V0	2017	[96]
PP	PEI-PA	20 wt.%	-0.6	25.1	-	2014	[97]
PP	PIP-PA	18 wt.%	61.6	25	V0	2019	[98]
PLA	PA	20 wt.%	31.0	-	V2	2017	[99]
PLA	PA/lignin	20 wt.%	44.0	-	V2	2017	[99]
PLA	PA-CaMg/CNT	20 wt.%	35.0	-	-	2018	[94]
PLA	PA-Na/PA-Fe	20 wt.%	47.0	-	V2	2015	[100]
PLA	PA/Urea-Co	20 wt.%	35.3	31.2	V0	2023	[101]
PVC	Sn–Phyt	15 wt.%	35.2	30.3	-	2018	[102]
PVC	Cu–Phyt	15 wt.%	44.9	29.3	-	2018	[102]
HDPE	IFR/Zr-PA	25 wt.%	46.4	23.5	N.R.	2020	[103]
PA6	PA-MEL-MWCNTs	7 wt.%	30.3	26.4	V0	2022	[104]
WPCs	PATA/APP	25 wt.%	49.0	25.0	V0	2022	[105]
EP	GPP@HNTs	10 wt.%	32.8	33.5	V0	2024	[106]
SPE	SPE-PA	7.7 wt.%	42.3	24.0	V0	2024	[107]
SPE	SPE-PA/MEL	5 wt.%	57.6	31.0	V1	2024	[107]
SPE	SPE-PA/APP	9.6 wt.%	80.8	55.0	V0	2024	[107]

Note: PP refers to polypropylene; PLA refers to polylactic acid; PVC refers to polyvinyl chloride; HDPE refers to high-density polyethylene; PA6 refers to polyamide 6; PEI refers to polyethylenimine; PIP refers to piperazine; SPE refers to sorbitol polyglycidyl ether epoxy resin; N.R., no rating.

### 3.5. Other Bio-Derived Flame Retardants

Beyond the extensively studied biomass-based flame retardants discussed earlier, other bio-derived FRs, such as starch, sodium alginate, tannic acid, proteins, and others, have also attracted considerable research interest [108,109,110,111,112,113,114,115,116]. These materials are being explored for their potential to offer sustainable, non-toxic, and effective flame-retardant properties, making them promising alternatives to conventional synthetic FRs.

Starch, a naturally occurring polymer found in plants, is composed of two primary macromolecules: amylose and amylopectin. Amylose is a linear polymer made up of glucose units, while amylopectin is a branched polymer. Both contribute to the ability of starch to store energy in plant cells. Chen et al. [117] synthesized a starch-based phosphorus and nitrogen synergistic flame retardant (SAPU) using corn starch as a carbon source, which was applied to cellulosic paper through impregnation. The results showed that the ring crush strength of the treated cellulosic paper increased by 156.2%, while the LOI reached 39.4%, demonstrating the effectiveness of the starch-based flame retardant in enhancing fire resistance.

**Figure 5:**
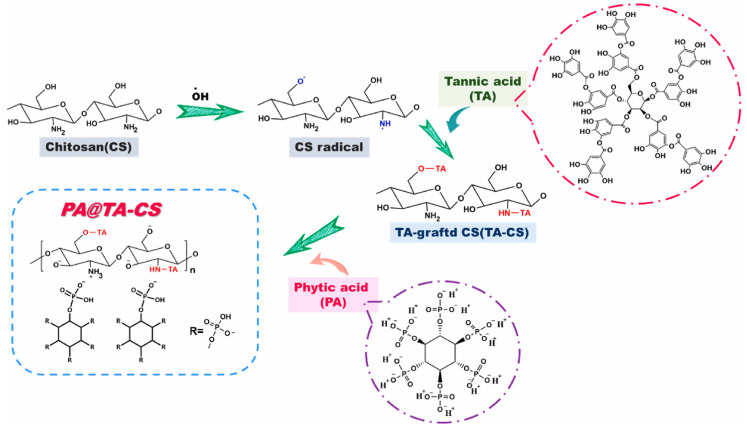
The synthetic route of PA@TA-CS. Copyright 2022. Reproduced with permission from Elsevier Science Ltd.

Tannic acid (TA) is a naturally occurring phenolic compound that is typically synthesized by plants as part of their secondary metabolism [118]. Tannins, the broader class of compounds to which TA belongs, are generally classified into two main categories: hydrolyzed tannins and condensed tannins. Hydrolyzed tannins consist of sugar moieties bonded to phenolic acids, while condensed tannins are composed of flavonoid units linked together, forming a more complex structure. Both types of tannins are recognized for their antioxidant, antimicrobial, and astringent performance, making them valuable in various industrial applications, including as potential bio-based flame retardants. As described in Figure 5, Qiu et al. [119] developed an entirely bio-based additive (PA@TA-CS) through a free radical grafting reaction and electrostatic adsorption, which forms an intumescent flame-retardant system via hydrogen bonding. In their study, the addition of 3% PA@TA-CS significantly enhanced the flame retardancy of PLA, achieving a LOI value of 26.9% and meeting the V-0 level in the UL-94 flame retardancy test.

Combining bio-based FRs with other fillers is an effective approach to improve the flame retardancy and overall performance of polymer composites. By incorporating fillers such as inorganic materials, nanomaterials, or natural fibers, the cooperative effect between the flame retardants and the fillers can enhance the thermal stability, mechanical properties, and flame resistance of the resulting materials. The use of fillers like clay, silica, or cellulose, in combination with bio-based FRs, can establish a more robust and efficient flame-retardant system by enhancing dispersion, improving thermal stability, and minimizing the generation of flammable gases during combustion. Montmorillonite was incorporated into TA to improve the fire safety of PLA [120]. Incorporating 17 wt.% tannic acid and 3 wt.% montmorillonite (TA/OMMT) into PLA resulted in a 50% reduction in pHRR and a 9.4% reduction in THR. The cooperative effect of OMMT and TA notably improve the carbon residues and enhanced the flame resistance of the PLA composites.

## 4. Surface Functionalization of Bio-Derived Flame Retardants

### 4.1. Surface Coating Through Layer-by-Layer Self-Assembly

Surface functionalization of bio-derived FRs is a growing area of research aimed at enhancing the flame retardance and overall performance of bio-based materials [121,122,123]. One effective approach involves the use of layer-by-layer (LbL) self-assembly technology [60,124,125]. This technique facilitates the precise deposition of multiple functional layers onto the surface of flame-retardant particles, resulting in the development of innovative FRs with core–shell structures. The core–shell structure enhances both the dispersion of the FRs within the polymer matrix and its stability, compatibility, and flame-retardant efficiency.

**Figure 6:**
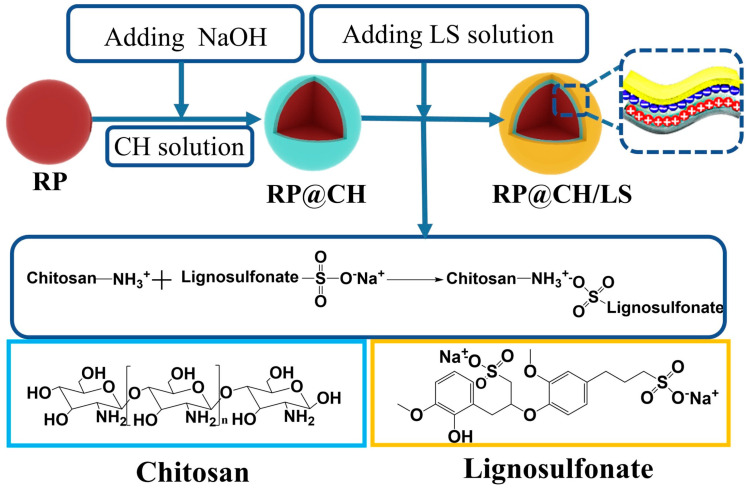
The preparation processes of RP@CH and RP@CH/LS [126]. Copyright 2021. Reproduced with permission from Elsevier Science Ltd.

**Figure 7:**
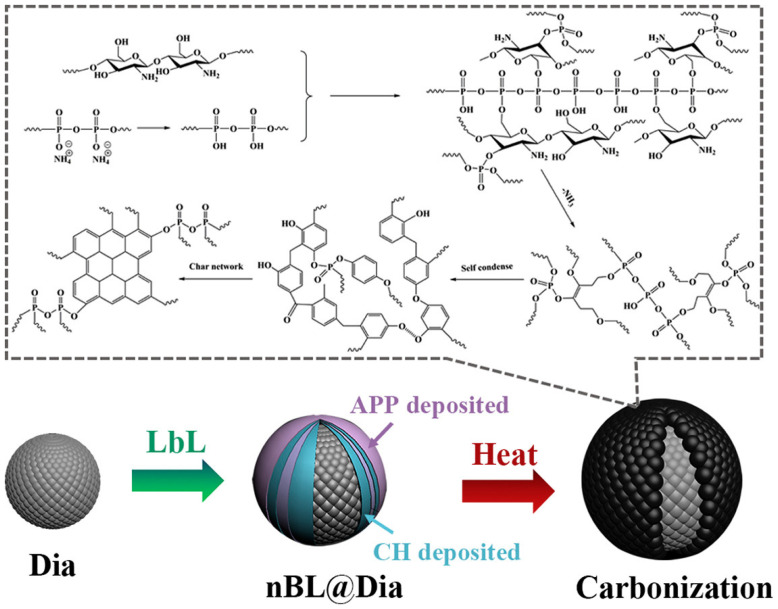
The flame-retardant mode of action of nBL@Dia in UPR composites involves the formation of a protective char layer through the decomposition of APP, CH and Dia [127]. Copyright 2020. Reproduced with permission from Elsevier Science Ltd.

As depicted in Figure 6, a novel core–shell flame retardant (RP@chitosan/LS) was developed, with microencapsulated red phosphorus (RP) as the core and chitosan and lignosulfonate (LS) as the shell [126]. At a 7 wt.% addition of this flame retardant, the LOI of the epoxy (EP) composite increased from 20.4% to 30.6%, and the composite achieved a V0 rating in the UL-94 test. Compared to the pure EP, the pHRR of the EP composites decreased by 59.8%, and the total heat release (THR) dropped by 48.2%. The core–shell structure enhances the catalytic formation of carbon during combustion, generates inert gases and effectively suppresses the combustion process. As illustrated in Figure 7, a novel flame-retardant system was developed by coating diatomite (Dia) with a composite of chitosan and ammonium polyphosphate (APP), which was then applied to unsaturated polyester resin (UPR) [127]. Compared to pure UPR, the pHRR of the composite material with a 9BL chitosan/APP@Dia coating decreased significantly, from 450.9 kW/m^2^ to 266.9 kW/m^2^.

### 4.2. Incorporation of Metal Ions to Bio-Derived Flame Retardants

The formation of salts with metal ions is a promising approach in the development of flame retardants, particularly when combined with other flame-retardant systems. Metal salts, often derived from metals such as aluminum, magnesium, or zinc, can act synergistically with other flame retardants, enhancing their effectiveness by improving char formation, reducing heat release and combustible gases during combustion.

A new core–shell flame retardant was developed using zinc phytate (PA-Zn) and two-dimensional graphite C_3_N_4_ (g-C_3_N_4_) as raw materials through a layer assembly method [128]. The EP/g-C_3_N_4_/PA-Zn composites were created by blending the flame retardant with epoxy resin (EP). The incorporation of PA-Zn facilitates the degradation of EP, leading to increased carbon residue formation during combustion. As a result, the combination of g-C_3_N_4_ and PA-Zn enhances the flame resistance and smoke suppression properties of the EP composites during the combustion process.

**Figure 8:**
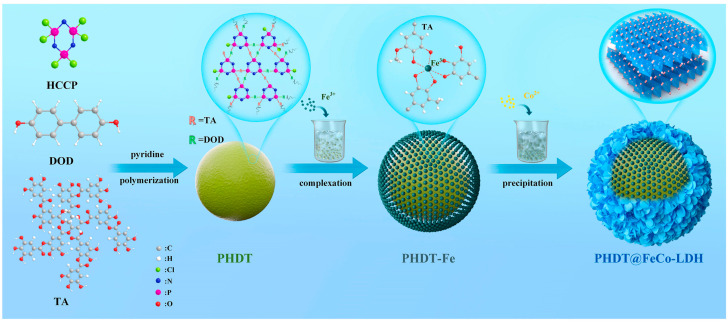
Preparation route of PHDT@FeCo-LDH. Copyright 2022. Reproduced with permission from Elsevier Science Ltd.

As portrayed in Figure 8, polyphosphazene microspheres (PHDTs), synthesized by condensing tannic acid and 4,4′-dihydroxybiphenyl, were modified with LDH to create the flame-retardant PHDT@Co-LDH [129]. When 4 wt.% of this novel flame retardant was incorporated into EP, the LOI value of the composite increased to 29.7%, and the UL-94 rating reached V0. The addition of this flame retardant significantly reduced both the pHRR and THR of the EP composite, while the FRI improved to 2.22. The presence of PHDTs promotes the carbonization of the EP matrix and helps capture radicals, thereby terminating the combustion reaction.

### 4.3. Conjunction with Other Flame Retardants

In the pursuit of enhancing flame retardancy, the combination of bio-based FRs with other conventional or synergistic flame retardants has emerged as a promising approach. This strategy capitalizes on the complementary mechanisms of various flame retardants, combining their unique properties to achieve superior fire resistance, reduced smoke production, and enhanced material performance. Specifically, the cooperative effects of boron-phosphorus, boron-silane, and metal oxide systems have shown significant promise in bio-derived flame retardants. Boron-phosphorus and boron-silane hybrid systems can enhance the development of a protective carbon-rich layer and the emission of non-combustible gases, effectively suppressing the combustion process and reducing smoke emissions [130,131]. Additionally, the incorporation of metal oxides such as cuprous oxide (Cu_2_O) [50], manganese dioxide (MnO_2_) [55,132], and magnesium oxide (MgO) [133] further boosts flame retardancy. The incorporation of MnO_2_ nanosheets and UiO66-NH_2_ to carbon fiber (CF) epoxy composites enhanced both mechanical and flame-retardant properties [55]. Specifically, the flexural and impact strengths increased by 50.9% and 23.8%, respectively, while pHRR and THR decreased by 54.99% and 78.65%, respectively. These improvements result from the enhanced fiber surface roughness and active groups on the fiber, while also acting as a physical barrier and catalyzing the formation of a dense char layer to hinder heat and smoke release effectively. The incorporation of magnesium oxide (MgO) microcapsules into cellulose fibers significantly improved their flame resistance and thermal stability [133]. Compared to unmodified cellulose fibers, the limiting oxygen index (LOI) value of cellulose-Mg fibers increased from 19 to 35, demonstrating enhanced flame retardancy. Thermogravimetric analysis (TGA) showed that the residue yield increased from 13.79% in unmodified fibers to 30.26% in cellulose-Mg fibers, highlighting the superior thermal stability imparted by the MgO microcapsules. Furthermore, when metal oxides are combined with boron, such as in boron-containing metal oxide composites, their synergistic interaction improves the material’s fire resistance by reducing flammable gas formation and enhancing the overall thermal stability. By incorporating these synergistic flame retardants, bio-derived systems can achieve more efficient, environmentally friendly flame protection, offering a significant advancement in fire-resistant bio-based materials.

Lignin-intercalated montmorillonite (LM) can serve as a synergist with intumescent flame retardants (IFR) in polybutylene succinate (PBS) composites [129]. When 22 wt.% IFR and 3 wt.% LM are incorporated, the sample achieves an LOI value of 36.5%. The addition of LM enhances the mechanical properties of the PBS composite, with 3% LM showing particularly strong results. In the presence of ammonium polyphosphate (APP), montmorillonite and lignin contribute to the formation of an expanded silicate carbon layer, while lignin releases a significant amount of inert gas during combustion. The combined action of LM and IFR substantially improves the flame-retardant performance and overall efficiency of the composite.

### 4.4. Life Cycle Assessment (LCA) Analysis

Life cycle assessment (LCA) is a critical tool in environmental management, designed to evaluate the environmental impacts (EIs) associated with a product throughout its entire life cycle [134,135]. It provides a comprehensive analysis of the environmental footprint from the extraction of raw materials through to production, usage, and final product disposal. This assessment includes all stages of a product’s life, such as transportation, manufacturing processes, and the impact of its use, thereby offering a holistic view of its environmental performance [136]. LCA aims to identify the cumulative environmental impacts that occur at each stage of the product’s life, from the sourcing of raw materials to the product’s end-of-life disposal. It allows for the evaluation of factors such as resource depletion, energy consumption, water usage, greenhouse gas emissions, and waste generation. This information is invaluable in making informed decisions about the design, production processes, and sustainability of products.

Jonkers et al. [137] carried out a comprehensive comparative life cycle assessment (LCA) that offers important insights into the environmental implications of various flame retardants. Their study concludes that halogen-free flame retardants (HFFRs), especially non-brominated types, present a significantly lower environmental impact when compared to traditional brominated flame retardants (BFRs). The analysis emphasizes that while BFRs are highly effective in providing fire resistance, they pose considerable environmental risks throughout their life cycle. The study underscores the urgent need for alternatives to BFRs, highlighting the environmental benefits of HFFRs in reducing pollution and minimizing ecological damage. Moreover, the findings suggest that transitioning to more sustainable flame-retardant options could help mitigate the environmental footprint of materials that require fire resistance, supporting the shift towards greener, safer industrial practices.

The importance of different phases of the life cycle of FRs in LCA studies is closely interconnected with their environmental exposure. The process of evaluating the environmental impacts of flame retardants encompasses all stages of their life cycle, from raw material extraction, production, and use to disposal or recycling. Each phase of this cycle contributes uniquely to the overall environmental impact, with varying degrees of environmental exposure. For instance, the production phase often involves significant energy consumption and emissions, while the end-of-life phase raises concerns about the release of hazardous substances during disposal or incineration. In LCA studies, understanding how these stages contribute to the environmental footprint of FRs is crucial in developing sustainable alternatives. Broeren et al. [138] present a comprehensive case study aimed at identifying bio-based alternatives to petrochemical plastics traditionally used in the production of flame-retardant panels. On a per-kilogram basis, bio-based plastics demonstrate 15–60% lower cradle-to-grave greenhouse gas (GHG) emissions and 10–60% lower non-renewable energy use (NREU) compared to petrochemical plastics. Additives contribute notably to impacts, particularly in bio-based FR grades, where they account for 5–25% of GHG emissions in low-end estimates and 10–45% in high-end estimates. For bio-based panels, despite a 20% weight increase to meet performance requirements, they maintain lower GHG emissions (1.7–2.5 kg CO_2_ eq.) compared to petrochemical panels (2.3–3.3 kg CO_2_ eq.), albeit with higher land use (0.8–2.1 m^2^·yr/panel). End-of-life incineration also contributes 35–60% of total emissions, but the lower carbon content of bio-based materials further reduces their overall environmental footprint. The study utilized a cradle-to-grave LCA approach, which evaluates the environmental performance of the candidate materials throughout their entire life cycle, from raw material extraction to disposal. Specifically, the LCA compared the environmental impacts of these bio-based materials to those of reference materials, focusing on key factors such as greenhouse gas emissions, non-renewable energy consumption, and agricultural land use per kilogram of material produced. The findings from this study highlighted that bio-based plastics not only match the environmental performance of conventional petrochemical plastics but, in some cases, even surpass them.

The increasing demand for environmentally sustainable materials in the polymer industry has led to growing interest in bio-derived FRs, which present a promising alternative to traditional halogenated and phosphorus-based retardants [139]. For the TPs-modified lignocellulose aerogel (TLA-4), the traditional FR systems often rely on halogenated or phosphorus-based chemicals, which are associated with higher energy consumption, greenhouse gas emissions, and toxicity in their production and disposal stages. In contrast, TLA-4, developed using a recyclable deep eutectic solvent (DES) and natural tourmaline particles (TPs), achieved superior flame retardancy, reducing the total heat release (THR) and heat release rate (HRR) by 62.0% and 66.3%, respectively, while enhancing the limiting oxygen index (LOI) by 74.1% compared to TPs-free aerogels. The environmental assessment revealed that TLA-4 had a lower environmental impact across 17 LCA categories, including reduced carbon emissions and energy consumption, compared to conventional aerogels. Additionally, TLA-4’s reliance on renewable lignocellulose, recyclable deep eutectic solvent (DES), and the release of negative oxygen ions (NOIs) during use further reduced its ecological footprint, making it a more sustainable alternative to traditional FR materials. In this context, conducting an LCA allows for the evaluation of the sustainability of these materials, considering their origin (such as plant-based or waste materials), production process, usage, and eventual disposal. By providing a comprehensive picture of a product’s environmental impact, LCA plays a vital role in the development of more sustainable and eco-friendly alternatives for polymeric materials.

## 5. Concluding Remarks and Future Aspects

The application and development of bio-derived flame retardants (FRs) offer significant opportunities, particularly in advancing sustainability and reducing environmental impact. Derived from renewable and natural resources such as lignin, tannins, and starch, bio-derived FRs present a promising alternative to traditional synthetic flame retardants, which are often toxic and environmentally harmful. These bio-derived materials are biodegradable, non-toxic, and can be sourced from abundant, low-cost raw materials, making them highly attractive for industries seeking greener alternatives. Moreover, bio-derived FRs can be used in conjunction with conventional flame retardants to create cooperative effects, resulting in enhanced flame resistance, smoke suppression, and mechanical properties. This allows for improved safety and performance in a variety of applications, including textiles, construction materials, and automotive products.

However, there are several challenges in the development and application of bio-derived FRs. One of the primary hurdles is the relatively low flame retardancy efficiency of many bio-derived FRs, which often require higher concentrations to match the performance of synthetic counterparts. This can adversely affect the mechanical properties of the base materials and raise the overall cost. Additionally, the compatibility of bio-derived FRs with different polymer matrices can be an issue, as they may not easily integrate with all materials. There are also concerns about the scalability and consistency of raw material sources, as the quality of bio-derived FRs can vary depending on factors like extraction methods and plant species. Moreover, bio-derived flame retardants can be more susceptible to degradation under environmental conditions, including UV exposure, moisture, and high temperatures. This can limit their long-term effectiveness in some applications. The development of more durable, weather-resistant bio-derived FRs, or the use of protective coatings, will be essential for expanding their application. A key aspect of promoting bio-derived flame retardants lies in understanding their environmental impact through a comprehensive life cycle assessment (LCA). By assessing the environmental footprint of bio-derived FRs throughout their life cycle from raw material extraction, production, and application to disposal or recycling. The renewable nature of bio-derived materials offers inherent advantages, such as reduced carbon emissions and minimized dependency on non-renewable resources. However, the LCA should also consider factors like energy consumption during processing, the carbon footprint of transport, and the end-of-life disposal or biodegradability of bio-derived FRs in various applications.

Despite these challenges, ongoing research is focused on improving the performance and stability of bio-derived flame retardants, such as through modifications, hybrid systems, and better incorporation techniques. Future innovations may address issues related to cost, scalability, and compatibility, making bio-derived FRs more competitive with traditional options. In the long run, bio-derived FRs hold the potential to provide safer, more sustainable fire protection solutions across a wide range of industries.

## Figures and Tables

**Table 1:** Comparison of LOI and UL-94 test results for flame-retardant composites with different lignin systems.

Composition of Materials	Methods	Fillers Content	PHRR Reduction (%)	LOI (%)	UL-94 Rating	Published Year	Ref.
PLA/lignin	FR directly	5 wt.%	1.3	-	V2	2016	[75]
PA 11/lignin	FR directly	20 wt.%	50.8	-	V1	2017	[76]
PP/lignin/APP	Synergistic FR	20 wt.%	71.4	-	-	2003	[73]
PLA/lignin/MCAPP/OMMT	Synergistic FR	23 wt.%	79.8	35.5	V0	2013	[77]
PLA/APP/lignin/OMMT	Synergistic FR	20 wt.%	52.5	39	V2	2022	[78]
EP/HL/DTZ	Synergistic FR	20 wt.%	50.2	36.6	V0	2024	[79]
PBS/P-lignin	P-Lignin	20 wt.%	47.5	-	-	2015	[80]
ABS/phosphorated lignin	P-Lignin	30 wt.%	58.0	-	-	2016	[81]
PLA/urea-modified lignin/APP	N-Lignin	23 wt.%	74.8	34.5	V0	2012	[82]
PP/PN-lignin	P and N-Lignin	30 wt.%	74.0	-	-	2012	[83]
PP/PN-lignin-Ni	P and N-Lignin	20 wt.%	75.6	26	-	2012	[84]
PLA/PN-lignin	P and N-Lignin	20 wt.%	33.0	-	V0	2016	[85]
ABS/Lignin-N-P NPs	P and N-Lignin	8.0 wt.%	67.9	-	-	2021	[86]
EP/Lig-HCCP	P and N-Lignin	20 wt.%	58.9%	28.2	V0	2022	[87]
PLA/Lig-Si/APP	Si-Lignin	20 wt.%	65.1	34	V0	2012	[88]
Cotton/lignin-silica-based FR/DOPO	Si-Lignin	-	78%	27.5	-	2024	[89]

Note: PP refers to polypropylene; PLA refers to polylactic acid; PVC refers to polyvinyl chloride; ABS refers to acrylonitrile butadiene styrene; EP refers to epoxy resin; PBS refers to polybutylene succinate.

## Data Availability

Not applicable.
